# Health facility barriers to HIV linkage and retention in Western Kenya

**DOI:** 10.1186/s12913-014-0646-6

**Published:** 2014-12-19

**Authors:** Juddy Wachira, Violet Naanyu, Becky Genberg, Beatrice Koech, Jacqueline Akinyi, Regina Kamene, Samson Ndege, Abraham M Siika, Sylvester Kimayo, Paula Braitstein

**Affiliations:** Moi University School of Medicine, P.O Box 4604–30100, Eldoret, Kenya; Academic Model Providing Access to Healthcare (AMPATH) Partnership, P.O Box 4604–30100, Eldoret, Kenya; Moi University School of Public Health, P.O Box 4604–30100, Eldoret, Kenya; Indiana University School of Medicine, 1110 W Michigan St, Indianapolis, IN 46202 USA; University of Toronto, Dalla Lana School of Public Health, 155 College St, Toronto, ON M5T 3 M7 Canada; Department of Health Services, Policy & Practice, Brown University, Box G-S121-7, 121 South Main Street, Providence, RI 02912 USA

**Keywords:** HIV, Linkage, Retention, Healthcare, Barriers, Social ecological model, Kenya

## Abstract

**Background:**

HIV linkage and retention rates in sub-Saharan Africa remain low. The objective of this study was to explore perceived health facility barriers to linkage and retention in an HIV care program in western Kenya.

**Methods:**

This qualitative study was conducted July 2012-August 2013. A total of 150 participants including; 59 patients diagnosed with HIV, TB, or hypertension; 16 caregivers; 10 community leaders; and 65 healthcare workers, were purposively sampled from three Academic Model Providing Access to Healthcare (AMPATH) sites. We conducted 16 in-depth interviews and 17 focus group discussions (FGDs) in either, English, Swahili, Kalenjin, Teso, or Luo. All data were audio recorded, transcribed, translated to English, and a content analysis performed. Demographic data was only available for those who participated in the FGDs.

**Results:**

The mean age of participants in the FGDs was 36 years (SD = 9.24). The majority (87%) were married, (62.7%) had secondary education level and above, and (77.6%) had a source of income. Salient barriers identified reflected on patients’ satisfaction with HIV care. Barriers unique to linkage were reported as quality of post-test counseling and coordination between HIV testing and care. Those unique to retention were frequency of clinic appointments, different appointments for mother and child, lack of HIV care for institutionalized populations including students and prisoners, lack of food support, and inconsistent linkage data. Barriers common to both linkage and retention included access to health facilities, stigma associated with health facilities, service efficiency, poor provider-patient interactions, and lack of patient incentives.

**Conclusion:**

Our findings revealed that there were similarities and differences between perceived barriers to linkage and retention. The cited barriers reflected on the need for a more patient-centered approach to HIV care. Addressing health facility barriers may ultimately be more efficient and effective than addressing patient related barriers.

## Background

Kenya is among the sub-Saharan countries that have embraced strategies such as HIV home-based counseling and testing (HBCT) [1,2] in an effort towards ‘zero’ new infections. In addition improved HIV treatment coverage in the region has been noted [3]. This has resulted in positive health outcomes including increased testing coverage and timely engagement in care among those found to be HIV positive [3,4]. Unfortunately, studies conducted in the region show that the rates of linkage to [3,5,6] and retention in HIV care, remain low [3,7,8]. Linkage, the timely entry into HIV care following HIV diagnosis, is reported to be less than 50% while retention, the continuous engagement of patients in HIV care, has been shown to decrease from 86% at 12 months to 72% at 60 months [3,7,8].

To effectively address the challenges of timely and continuous engagement of HIV patients in care, there is need for better understanding of the potential barriers across various socio-cultural environments. Previous studies have reported factors such as being male, younger in age, fears of drug side effects, busy schedules, transport costs and distance, stigma and fear of disclosure, staff shortages, and delays at the health facility as contributing to poor linkage and retention in the region [3,9-11]. However, few studies have specifically focused on how the structural features of health facilities present barriers to patient engagement in HIV care in sub-Saharan Africa [12]. We believe that health facility factors may be the most amenable to intervention compared to patient factors such as transport cost, HIV stigma and fear of disclosure. Addressing factors at the structural level may also alter the socio-cultural context and result in changes to patient perceptions and attitudes regarding HIV care.

We therefore investigated patients’, caregivers’, and health care providers’ perspectives on HIV linkage and retention. We focused on communities within the Academic Model Providing Healthcare (AMPATH) program in western Kenya that provides a wide range of healthcare services including HIV prevention, treatment, and care [13]. The specific objective of this study was to describe perceived healthcare system-level barriers to linkage and retention.

## Methods

### Study setting

AMPATH was initiated in 2001 as a joint partnership between Moi University School of Medicine, the Indiana University School of Medicine, and the Moi Teaching and Referral Hospital. The initial goal of the program was to establish an HIV care system to serve the needs of both urban and rural patients as well as to assess the outcomes and barriers of ART. Over the years, AMPATH has expanded to embrace primary healthcare and chronic disease management. The program has enrolled more than 150,000 HIV-infected adults and children in >65 Ministry of Health facilities and numerous satellite clinics (outreach clinics) across western Kenya. All HIV and tuberculosis-related care and treatment are provided free at initiation of care. This study was undertaken in three AMPATH sites, namely Turbo, Teso, and Chulaimbo.

### Target population

This study targeted patients within the AMPATH program including patients receiving HIV, TB, and hypertension care, as well as caregivers of children with HIV, community leaders (religious leader, traditional healer, village elder, assistant chief), and healthcare workers, namely volunteer community health workers, the AMPATH safety net team (Nutritionist, Psychosocial, Outreach, Social work teams) and, healthcare providers (AMPATH clinical team, Ministry of Health staff).

### Study design

This qualitative study was conducted between July 2012 and August 2013. Study participants were purposively sampled from the three AMPATH sites. In-depth interviews and focus group discussion (FGDs) were used to collect data. We adopted the social ecological model, previously used to explore engagement in HIV care at multiple levels including the intrapersonal, interpersonal, organizational, community and policy levels [12].

### Human subjects protection

Ethical approval was obtained from the Moi Teaching and Referral Hospital Institutional Research and Ethics Committee (IREC) as well as the Indiana University Institutional Review Board (IRB). Oral consent was obtained from all participants. All interview sessions were conducted in private rooms. Privacy and confidentially were assured at all times. During all interview sessions first names were used to facilitate discussions.

### Study procedure

We conducted a total of 16 in-depth interviews and 26 FGDs. However for this study we focused on 16 in-depth interviews and 17 FGDs comprising of an average of 7 participants per FGD that centered on structural barriers to HIV linkage and retention. Table 1 shows the distribution of interview sessions per site. A set of interview guides were developed to explore perceived barriers at the various social ecological levels. In addition, basic socio-demographic information of age, gender, educational level and occupation was only collected for those who participated in the FGDs. The guides were then translated to Swahili, Kalenjin, and Luo. Trained research assistants identified the target groups at AMPATH health facilities and informed them about the study. Health facilities in-charges assisted with contacting HIV patients who attended the AMPATH HIV clinics. Those willing to participate in the study were referred to research assistants who were stationed in private rooms. Oral consent was obtained from all participants. The interview sessions took approximately 1 hour and were conducted in either, English, Swahili, Kalenjin, or Luo. All sessions were audio recorded and for the FGDs, scribes also recorded session proceedings. At the end of each session participants were provided with transport reimbursement of Kenya Shillings (Ksh) 200.

**Table 1:** **Distribution of interview sessions by site**

**In-depth interview sessions**				
**Site**	**Patients**	**Caregivers**	**Community leaders**	**Healthcare workers**
Teso	-	-	3	2
Chulaimbo	-	-	4	2
Turbo	-	-	3	2
**FGD sessions**				
**Site**	**Patients**	**Caregivers**	**Community leaders**	**Healthcare workers**
Teso	4	1	-	3
Chulaimbo	3	-	-	3
Turbo	1	1	-	1

### Data analyses

Recorded interviews were transcribed and translated to English. The data were then coded and themes related to barriers to HIV linkage and retention were identified. Ideas from different interviews were then pooled together and integrated into common themes. Concepts from these themes were generated and used to organize the presentation of the results. For validation, independent coding and identification of themes were conducted by five investigators. The final write up consisted of summaries, interpretations and textual excerpts.

## Results

A total of 150 participants including; 59 patients diagnosed with HIV, TB, or hypertension; 16 caregivers; 10 community leaders; and 65 healthcare workers participated in the study. Table 2 shows the characteristics of participants in the FGDs. The mean age was 36 years (SD = 9.24). The majority (87%) were married, (62.7%) had a secondary level of education and above, and (77.6%) had a source of income.

**Table 2:** **Characteristics of participants in the FGDs**

**Characteristic**	**N = 134**	**100%**
	**n**	**%**
Gender		
Female	78	58.2
Male	56	41.8
Marital Status		
Single	22	16.4
Married	87	64.9
Separated/Widowed	13	9.7
Missing	12	9.0
Education Level		
None	6	4.5
Primary	44	32.8
Secondary	36	26.9
Tertiary	48	35.8
Occupation		
None	30	22.4
Casual	13	9.7
Formal	49	36.5
Self-employed/Farming	42	31.3

### Health facility barriers to hiv linkage and retention

**Figure 1:**
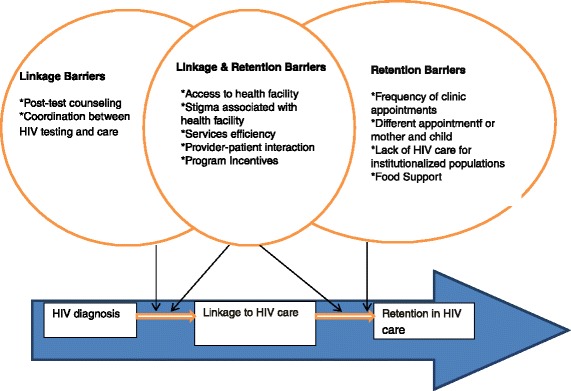
**Barriers to Linkage and Retention in HIV Care.**

A number of health facility factors emerged as salient barriers to linkage and retention. There were minimal differences across the study groups. Figure 1 highlights cited barriers that were unique to and similar for linkage and retention.

### Barriers that were unique to linkage

#### Quality of post-test counseling

The lack of adequate time for post-test counseling at either the household or facility level was highlighted as a barrier to linkage. Time limitations were seen to compromise the quality of post-test counseling offered especially among pregnant women and discordant couples. These were further complicated by the lack of centralized care services where HIV patients were required to seek care in specialized HIV care clinics.

Healthcare worker (in-depth interview)- Teso*: They require a lot of counseling* (referring to discordant couples) *and the HBCT teams, I think did not have enough time to do that.*

Healthcare worker (FGD) -Teso: *Maybe let me talk about PMTCT for the pregnant mothers. At times you have come to the facility* (referring to antenatal care) *because you are pregnant, you are tested and found to be HIV positive….then you are told to go and enroll elsewhere* (PMTCT program within the health facility)*… Remember you have just learnt about your status and you don’t want so many people to know about it…so it becomes so difficult for them to go for HIV care.*

#### Coordination between HIV testing and care

Participants also mentioned that the health facilities lacked a proper link between HIV testing and care. HIV patients were reported to visit health facilities for initial care but returned home without being seen by a clinician because they were intimidated by the clinic layout and procedures.

Healthcare worker (FGD) -Turbo: *Because most of them disappear on the way, all the way from the voluntary counseling and testing (VCT) to the AMPATH clinic* (all in the same health facility) *there is some distance there and we need some other system… maybe have more staff or counselors so that one could provide counseling and the other one could escort newly diagnosed HIV patients to their clinic.*

Healthcare worker (in-depth interview) -Teso: *But the challenge was escorting patients to the HIV clinics. I remember a time when we had so many people who had tested HIV positive but when we looked at the enrollment, the figures were not tallying.*

### Barriers related to both linkage and retention

#### Health facility access

The distance to the health facility coupled with the poor terrain made it challenging for new and returning HIV patients to assess care. For retention this factor was associated with the health facility boundaries that defined facilities’ geographical coverage. Patients living along/close to health facility boundaries faced the greatest challenge. They were forced to seek care in facilities farther from their homes because those facilities were considered in-catchment.

Community leader-Chulaimbo: (Linkage) *This health facility covers even beyond that hill (shows the moderator) yet there is no accessibility. From that hill you have to go round because there is no road. If you go through Kima, it will take you two hours driving. So the easier way would be a direct route however, there is no road.*

Caregiver-Turbo: (Retention) *You are already in a place where there is no clinic yet Turbo-AMPATH clinic* (in-catchment) *is so far. Or you may be closer to a place like Webuye-AMPATH clinic* (out-of-catchment) *and it is obvious that when you go to Webuye-AMPATH clinic, they will ask for a transfer form from Turbo-AMPATH clinic. You cannot pick drugs from Webuye-AMPATH clinic.*

#### Stigma associated with the health facility

Since its inception AMPATH had focused on providing comprehensive HIV care to communities in western Kenya. Overall participants thought the program had played an important role in mitigating the spread of HIV as well as promoting the quality of life of communities living with HIV. This however meant that the program had a reputation of being an HIV care facility and given that HIV related stigma was still evident in surrounding communities, new and returning HIV patients did not want to be seen at these facilities.

HIV Patient-Chulaimbo: (Linkage) *There is bus stage in Kisumu that transports people to Chulaimbo-AMPATH clinic. Anybody seen at that stage is branded a sick person* (referring to HIV infected)*. People heading to Chulaimbo-AMPATH clinic for treatment are considered HIV infected people going for medicines…If you are seen at the Chulaimbo stage in Kisumu they say that this one is going to ‘Andila’ (luo word)…. ‘Andila’ is the name of these antiretroviral drugs that we take.*

Healthcare worker (FGD)-Turbo: (Retention) *If they are referred here* (referring to Turbo-AMPATH clinic), *there is still stigma associated with the AMPATH clinic…. that it is an HIV clinic. People will not want to associate themselves with AMPATH so they will definitely not come to the clinic for their next appointment. They know if they are seen there, it is like they are infected with HIV… They fear being seen by their neighbors.*

#### HIV drug package

Interestingly stigma associated with HIV drug packaging influenced patients’ care seeking behaviors. Participants reported that HIV drugs were packaged in big containers that were intimidating and stigmatizing at the same time. Hence some HIV patients opted not to seek or continue with care.

HIV patient -Chulaimbo: (Linkage) *The packaging of antiretroviral drugs… it gives people stress…for example the second line drug packaging is this big (demonstrates using the hands)…particularly when you are on your way home, you get stressed…when you are given drugs for two months, you look like you are from buying several items from the shop.*

Hypertension patient-Teso: (Retention) *Even packaging, there should be an improved way of packaging these antiretroviral drugs. The containers are too big and stigmatizing.*

#### Inefficient service delivery

The inefficiencies of health facilities such as delays in service delivery and long queues were raised as factors contributing to poor linkage and retention. Delays were also attributed to low staff to patient ratio and busy patient schedules. Perceptions about inefficiency of services were also influenced by inadequate knowledge about treatment procedures by patients and caregivers.

Healthcare worker (FGD) –Turbo: (Linkage) *When these HIV infected people come here (Turbo-AMPATH clinic), there is delay. Some of them get very discouraged. They say the staff are not attending to them well because may be they are in a hurry or want fast services so that they can go attend to their personal business. You know most of these HIV infected clients struggle to meet their basic needs. They want fast service so that they can find time to go and work and provide for their children. If they are delayed, they feel very bad and prefer private clinics for that provide efficient services.*

Caregiver-Teso: (Retention) *The long waiting for results. For me I stayed for nearly six months and every time I was told that the results are not yet out. This makes us always live in suspense.*

Healthcare worker (in-depth interview) -Chulaimbo: (Retention) *I can also add frustrations at the clinic …as we expand we realize that we have limited staff hence the waiting time for our patients increases. Some of them go through the frustration for a while then eventually give up.*

#### Patient-provider interaction

Lack of good provider-patient interaction was also cited as a barrier for both linkage and retention. There was mention of rude and unfriendly health providers who did not receive new patients well or empathize with returning patients when they missed a medical appointment. It was also reported that some patients received special privileges because they were related to the health providers. On the other hand, lack of confidentiality by healthcare providers was also raised as a concern. There was fear that health providers would disclose the HIV status of patients known to them.

Care givers-Turbo: (Linkage) *Maybe the services, people are not equal, others are quite emotional. If they come and realize that the services are slow, they might get upset and go away, never to return. They might go saying they weren’t given a warm reception, treated well, or even stayed in the queue for some hours… so new HIV clients might get discouraged to come for care.*

Care givers-Turbo: (Communication dynamics-Linkage) *There are those who start but stop along the way. In most cases the healthcare providers contribute because they talk badly. There was a time I defaulted on my treatment. I missed an appointment for my child (HIV infected) and when I came to the clinic the doctor was too harsh on me and said, “ You are the one who looked for this (referring to HIV infection)…” I went home and stayed for some years without coming for care. I became very ill then I had to come back to the clinic. So the manner in which the doctor talks to the patient makes a lot of difference.*

Care givers-Turbo: (Communication dynamics-Linkage)*: Same to me, there was a time I came on my appointment date. On reaching the clinic I was questioned about my drugs. I had travelled and I hadn’t carried my drugs. I was asked where my drug container was and I told them I had travelled and I had forgotten my drugs. They asked if I had gone to school (referring to being stupid). I was discouraged and upset. I left without taking the drugs. I felt like a prisoner.*

Community leader-Chulaimbo: *The only problem can be when others are attended to while you are kept waiting on the bench…For example if a health provider gives priority to his family member, you feel bad and go back home without being attended to.*

Healthcare worker (FGD) -Teso: (Retention-Confidentiality): *Ok some (health providers) are confidential but others are not. You will find a HIV client coming to the facility complaining that a certain health provider spoke about their HIV status to other community members. Now they are stigmatized.*

#### Program incentives

A number of health organizations were reported to be offering the same HIV care services as AMPATH. These health organizations were seen to be providing incentives such as transport, bed nets, and blankets, thereby luring patients to their health facilities. Hence, patients moved from one facility to the other in order to benefit from the incentives. In addition, the distance to some AMPATH health facilities made patients prefer other health facilities closer to them including dispensaries.

Community leader-Chulaimbo (Linkage): *Another thing is that there are organizations which provide other things like nets and blankets. Here (Chulaimbo-AMPATH clinic) people are only given food so there is a clash of interest.*

Healthcare worker (FGD) -Chulaimbo (Retention): *We have other facilities not only Chulaimbo-AMPATH clinic that are offering the same HIV care services for example dispensaries. People opt to go to the nearest health facilities.*

### Barriers specific to retention

#### Frequency of clinic appointments

To achieve the optimal benefits of HIV care, patients have to keep all their medical appointments. The frequency of clinic visits for follow-up care was cited as causing fatigue and thereafter loss to follow up. This was heightened by the distance to the health facility as well as busy patient schedules.

Healthcare worker (FGD) -Teso: *I would give an example of some patients coming once every week… it makes them tired…Yes, the frequency matters a lot.*

#### Different appointment for mother and child

Having different appointments for HIV positive mothers/caregivers and their children was mainly a challenge mentioned by caregivers. This was attributed to the cost implication of making several trips to the HIV clinic.

Caregiver-Chulaimbo: *It is challenging for me who comes from far. My child and I may have different clinic appointment dates. This requires that I look for money for transport to bring my child and myself to clinic on those separate days.*

#### Lack of HIV care service for institutionalized populations

The lack of specialized HIV clinics that served individuals who were institutionalized, for example children in schools and prisoners, was highlighted as a barrier to follow-up care. Stigma associated with disclosure of HIV status and amount of antiretroviral drugs were reported to hinder adherence to HIV care among patients in these institutions.

Healthcare worker (in-depth interviews) -Chulaimbo: *It is because the student, the parent or the caretaker has to disclose to at least one or two of the teachers to make sure the child takes medication. One cannot be given drugs for the whole school term so the parent or the caretaker has to take the drugs to school. When the other students get to know their HIV status, it becomes another issue…Let me give a picture of one student who defaulted and really needed a lot of counseling. He was in school and had his medication. Unfortunately, his fellow student teased him and told him that he had opened a pharmacy given the medication he was taking. Because of the way the other students perceived him he stopped coming for treatment.*

#### Food support

Over the years AMPATH with the support of the World Food Program (WFP), provided nutritional supplements to households of HIV infected patients assessed and found to be food insecure or malnourished. However recently, WFP changed the country strategy from feeding the patients’ household to only feeding the severely and moderately malnourished HIV patients. In addition the number of patients receiving food support was restricted to the very needy. This influenced retention rates because patients were unhappy with the new approach to food support, opting to dropout of care. It was also reported that in the effort to promote linkage, HBCT counselors assured HIV positive clients that they would get food support if they sought care. This was without consideration of the eligibility criteria for enrollment on the food program. When this expectation was not met, patients dropped out of care.

Caregivers-Teso (Lack of food): *Another thing is when some patients are given food and others are not, one can feel bad…. Yes the one who was not given food thinks that they are being discriminated upon.*

Healthcare worker (FGD) -Teso (End of food support): *This other support like the food program. If a person is weaned off the food program, he/she feels rejected. Hence they stop coming for HIV care.*

Healthcare worker (FGD) -Teso (Unmet expectations): *It also depends on the information that HIV testing counselors provide to HIV patients during linkage. You may emphasize so much on the food program of which the nutritionist may not agree with because you are not adhering to the eligibility criteria. That food support may have been the reason why the HIV positive patients came for HIV care in the first place.*

## Discussion

Engaging HIV patients in care, immediately after diagnosis and consistently thereafter, has a profound impact on efforts towards ‘zero new infection’ [3,5,7,8]. Similar to other studies [9,10,12], we noted a number of health facility barriers that influenced HIV care. Our study however adds to the existing literature by providing additional insight on structural barriers to linkage and retention. For example we reported barriers such as quality of post-test counseling, reception of new HIV clients, HIV drug packaging, lack of HIV care for institutionalized populations, and data discrepancies that have not been previously highlighted. The majority of barriers were reported for retention given the complex and long-term nature of HIV care and treatment. Overall barriers reflected on patients’ satisfaction with the quality of HIV care provided highlighting the need for a more patient-centered approach to HIV care that appreciates the role of the patient in care. Addressing health facility barriers may be one of the critical and primary steps in promoting linkage and retention.

The quality of post-test counseling after HIV diagnosis plays a critical role in defining patients’ care seeking behaviors [14]. This is constructed by the level of counselor training, workload, time, and support systems for counselors [14]. Testing programs such as HBCT provide a convenient platform where community members get tested for HIV at the household level. However, previous studies have reported that HBCT may be quite intensive, requiring extensive counseling skills and time in order to enhance linkage to care [2]. Our findings revealed that the lack of adequate post-test counseling influenced the preparedness of individuals to initiate care. Programs providing HIV testing should consider the time and quality of post-test counseling provided in order to adequately prepare HIV positive individuals for care. Post-test counseling should be an ongoing process that extends beyond diagnosis.

HIV linkage was hampered by the poor coordination between point of HIV testing and care. In recent years, the peer navigator system has been advocated as an approach to enhance patient engagement in HIV care [12,15]. Peer navigators are mostly HIV infected persons sharing similar socio-cultural environments as the patients. Their role is to enhance the awareness and utilization of available healthcare services while addressing some of the social-behavioral issues patients experience as they initiate or continue in care [12,15]. Health facilities in Kenya may need to adopt this approach as one of the mechanisms to promote linkage and retention.

As supported by our findings, previous studies have cited access to healthcare facilities as a barrier to care [8-10]. Interestingly our findings revealed that this was influenced by the health facility boundaries that restricted patients living along/close to the catchment borders. Furthermore stigma associated with HIV care facilities contributed to poor linkage and retention rates. This was further facilitated by the lack of centralized healthcare services requiring HIV patients to seek care in specific HIV stigmatized clinics. Decentralization of HIV care services right at the community level by ensuring all levels of government health facilities provide adequate HIV care services is critical to addressing this concern [16,17]. In addition, integration of care and treatment services across an array of diseases may be vital to eliminate discrimination of HIV patients in this setting. This should be combined with continuous HIV campaigns to address community stigma. However in doing so, there is need to redefine the health facility coverage protocols that restrict patients from receiving care in health facilities nearest to them.

Consistent with other studies in the region [9-11], our findings revealed various aspects associated with patient satisfaction with care such as delays at the clinic, long queues, favoritism, health provider’s attitudes, poor provider-patient communication, and lack of confidentiality among health providers as contributing to poor linkage and retention. Approaches such as integration of HIV in primary healthcare have been shown to improve patients’ satisfaction with care and ultimately care seeking behaviors [18]. However there is need to ensure that patient-centered approaches are put in place in order to fully engage patients in their care. There is also need to reevaluate the interpersonal skills and capacities of HIV care providers in view of the reported poor provider-patient interactions and the possible influx of new HIV patients following enhanced testing coverage [12].

To achieve the optimal benefits of HIV treatment, patients require consistent follow-up care. This necessitates making several clinic visits that may be overwhelming to some patients. In such cases peer navigators or case managers can be used to promote adherence to care [12]. Furthermore, as reported in a previous study [10], HIV infected caregivers caring for HIV infected children experienced challenges keeping all medical appointments. This was especially when the caregiver and child were given different appointment dates. The need to integrate adult and child HIV care in such situations is fundamental in order to address the physical and financial burden caregivers’ face.

AMPATH has been known for its comprehensive approach to HIV care that entails provision of nutritional supplements to food insecure and malnourished HIV patients and their entire household [13]. Counselors providing HIV testing were reported to entice HIV positive client to care by guaranteeing that they would receive food among other benefits, once in care. Unfortunately, the lack of or end of food support presented an obstacle to care. Patients preferred health facilities providing the best care incentive package which also meant relocating from one facility to another. This raises concern over the dependency on such support services. Health program will need to critically assess the sustainability of providing incentives prior to their implementation. There is also need for collaboration among existing HIV program to avoid competing interests that negatively influence patients’ care seeking behaviors.

Finally tailoring HIV care for specialized HIV populations including most at risk populations (MARPS) is vital to addressing the unique attributes within these groups. The lack of customized care for institutionalized populations mainly schools and prisons was viewed as barrier to retention. This was coupled with stigma associated with HIV, still eminent in these institutions. HIV programs need to consider the socio- cultural environment in which patients are embedded in and tailor care for them. In addition, continuous effort to address HIV stigma across institutions should be encouraged in order to provide conducive environments for HIV care and treatment. Interestingly, ARV drug packaging was also viewed as restricting linkage and retention due to stigma associated with the packaging. Health facilities and pharmaceutical companies will need to reevaluate the antiretroviral drug packaging in light of this finding. Providing less stigmatizing drug containers that are not only convenient to carry around but also enhance proper storage of drugs, is of essence.

Given the limited evidence-based health system level approaches recommended to promote linkage and retention in the region [12], our study provides insight on health facility barriers to HIV linkage and retention in Kenya that may be used to develop appropriate interventions. However our study is not without limitations. This was a qualitative study and we acknowledge that our findings cannot be generalized to the wider Kenyan population. It mainly presented the views of communities studied and was limited to their perceptions about AMPATH health facilities.

## Conclusion

In conclusion this study provides valuable insight on health facility factors reported as barriers to linkage and retention. There were similarities as well as unique differences in the barriers cited as influencing linkage and retention that mainly reflected on patients’ satisfaction of HIV care. We believe that this study is an important step towards understanding HIV care systems in Kenya and the challenges they pose for engaging patients in care. Our findings could also be used to guide the development of patient-centered HIV care programs that ultimately improve the rates of linkage and retention in the region.
